# The long head of the biceps tendon as a pedicled autograft for acromioclavicular joint reconstruction: development of an arthroscopic technique

**DOI:** 10.1007/s00276-026-03844-8

**Published:** 2026-03-13

**Authors:** Nuno Sevivas, Mariana Pinto, Diogo Nunes Sousa, Diogo Barreira, Ana Catarina Ângelo, Clara Azevedo, Manuel Ribeiro da Silva, Rui Claro, João Espregueira-Mendes, Hélder Pereira, Alexandre Lädermann

**Affiliations:** 1 Life and Health Sciences Research Institute (ICVS), School of Medicine, University of Minho, Campus de Gualtar, Braga, 4710-057 Portugal; 2 ICVS/3B’s - PT Government Associate Laboratory, Braga/Guimarães, Portugal; 3 Shoulder and Elbow Unit, Orthopaedics Department, ULSAM Médio Ave, Famalicão, Portugal; 4 Shoulder and Elbow Unit, Orthopaedics Department, Trofa Saúde Hospital Braga Sul, Braga, Portugal; 5 Shoulder and Elbow Unit, Department of Orthopaedic Surgery, Hospital dos SAMS de Lisboa, Lisbon, Portugal; 6 Shoulder and Elbow Unit, Orthopaedics Department, Joaquim Chaves Saúde, Carcavelos, Portugal; 7 Shoulder and Elbow Unit, Orthopaedic and Musculoskeletal Centre, Cuf Porto Hospital, Porto, Portugal; 8 Shoulder and Elbow Unit, Orthopaedics Department, Centro Hospitalar Santo António, Porto, Portugal; 9 Clínica Espregueira Mendes, Porto, Portugal; 10 Orthopaedics Department, Centro Hospitalar Póvoa de Varzim / Vila do Conde, Póvoa de Varzim, Portugal; 11 Ripoll y De Prado Sports Clinic: Murcia-Madrid FIFA Medical Centre of Excellence, Madrid, Spain; 12 Division of Orthopedics and Trauma Surgery, La Tour Hospital, Meyrin, Geneva, Switzerland; 13 Faculty of Medicine, University of Geneva, Geneva, Switzerland; 14 Division of Orthopedics and Trauma Surgery, Department of Surgery, Geneva University Hospitals, Geneva, Switzerland; 15 Foundation for Research and Teaching in Orthopedics, Sports Medicine, Trauma, and Imaging in the Musculoskeletal System, Meyrin, Geneva, Switzerland

**Keywords:** Shoulder surgery, Dislocation, Complication, Minimally invasive surgical procedure, Stability

## Abstract

Acromioclavicular joint (ACJ) dislocation is a frequent shoulder injury, and high-grade cases often require surgical reconstruction. Although multiple techniques have been described, no single method has achieved consensus as the gold standard. The long head of the biceps tendon (LHBT) represents an attractive autologous option, as it is anatomically adjacent to the ACJ, preserves vascularity when used as a pedicled graft, and reduces donor-site morbidity. Building on prior anatomical studies, this cadaveric investigation describes and validates an arthroscopic technique for ACJ reconstruction using the LHBT. The graft is mobilized while maintaining its supraglenoid origin, passed through a clavicular tunnel under endoscopic and fluoroscopic guidance, and reinforced vertically with a suspensory (suture-button) device passing independently through a coracoclavicular tunnel, and with an acromioclavicular cerclage to enhance horizontal stability. The procedure proved feasible, reproducible, and safe with respect to anatomical landmarks and adjacent structures.

**Clinical relevance**: This technique offers a biologically sound, minimally invasive alternative for ACJ reconstruction that may reduce morbidity and improve reproducibility compared with conventional graft-based procedures.

**Supplementary Information:**

The online version contains supplementary material available at 10.1007/s00276-026-03844-8.

## Introduction

Acromioclavicular joint (ACJ) dislocation is one of the most frequent traumatic lesions of the shoulder girdle, although its true incidence is probably underestimated in the general population [6]. Over the years, numerous surgical techniques have been proposed, including both anatomic and nonanatomic reconstructions. Despite these advances, no single approach has emerged as the definitive gold standard [18]. The choice of surgical strategy is influenced by several factors, including the grade and chronicity of the lesion, and the patient’s functional requirements [5]. Current biomechanical and clinical evidence supports anatomic reconstruction of both the acromioclavicular (AC) and coracoclavicular (CC) ligaments to restore physiological joint kinematics and stability [7, 15, 16, 20]. This can be achieved by using suspensory (suture-button) devices, tendon grafts, or hybrid constructs [5]. Moreover, when reconstruction is performed more than 10 days after injury, the use of a tendon graft appears necessary to optimize clinical outcomes [9].

The long head of the biceps tendon (LHBT) represents an attractive autologous option for biological augmentation. Its anatomical course—arising from the supraglenoid tubercle and superior labrum, traversing intra-articularly, and continuing extra-articularly within the bicipital groove—provides sufficient length and mobility for graft preparation. Importantly, the LHBT can be used as a pedicled autograft, thereby preserving its native vascularity and biological potential [8]. Furthermore, harvesting the LHBT avoids the donor-site morbidity associated with hamstring autografts or the risks linked to allograft use [21].

In a previous study, we reported the use of the LHBT as a pedicled autograft through an open approach, demonstrating its biological and mechanical potential for the treatment of chronic ACJ dislocations [21]. Building on these anatomical and surgical foundations, we hypothesized that the LHBT could serve as a pedicled autograft to augment ACJ reconstructions using an arthroscopically assisted procedure. This minimally invasive approach not only preserves the biological healing potential of the LHBT but also enables an anatomic reconstruction of both the CC and AC stabilizers, thereby providing combined vertical and horizontal stability. The present study outlines the anatomical rationale, developmental process, and sequential steps of this novel surgical technique.

## Surgical technique

This study was conducted in accordance with the Declaration of Helsinki and approved by the Ethics Committee of the Life and Health Sciences Research Institute – ICVS (reference number: CEICVS 171/2023). The procedure was developed and validated in the anatomy laboratory using fresh-frozen cadaveric specimen donated for research and educational purposes. Donors had provided informed consent prior to death, and specimens were handled in compliance with institutional and ethical standards. Each specimen was thawed at room temperature for 24 h before dissection.

### Patient setup

**Fig. 1:**
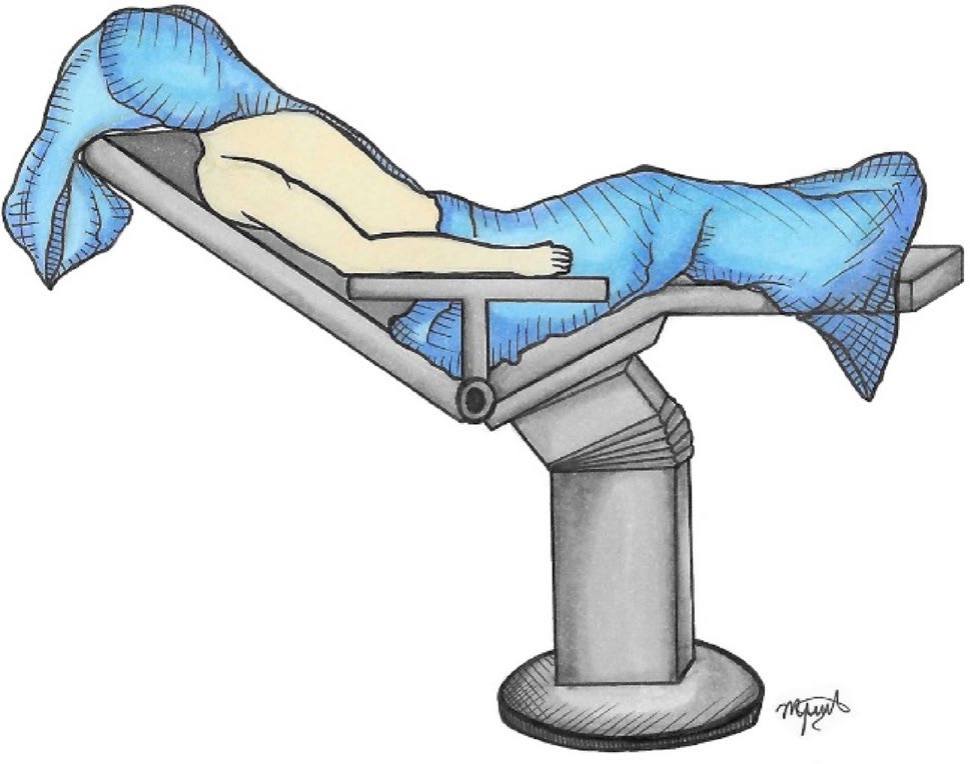
Patient positioning and surface anatomy. Beach-chair position with neutral arm support. Anatomical landmarks (clavicle, acromion, coracoid) marked prior to incision

The patient is placed in the beach-chair position with the affected arm resting neutrally at the side, optionally supported by a mechanical arm holder (Fig. 1). Before incision, the ACJ is reassessed under fluoroscopy for reducibility, and anatomical landmarks (clavicle, acromion, and coracoid) are carefully marked.

### Approach

In addition, a 3 cm mini-open incision is made over the ACJ to allow debridement, facilitate joint reduction, and expose the deltotrapezial fascia and ACJ capsule.

### Reduction

**Fig. 2:**
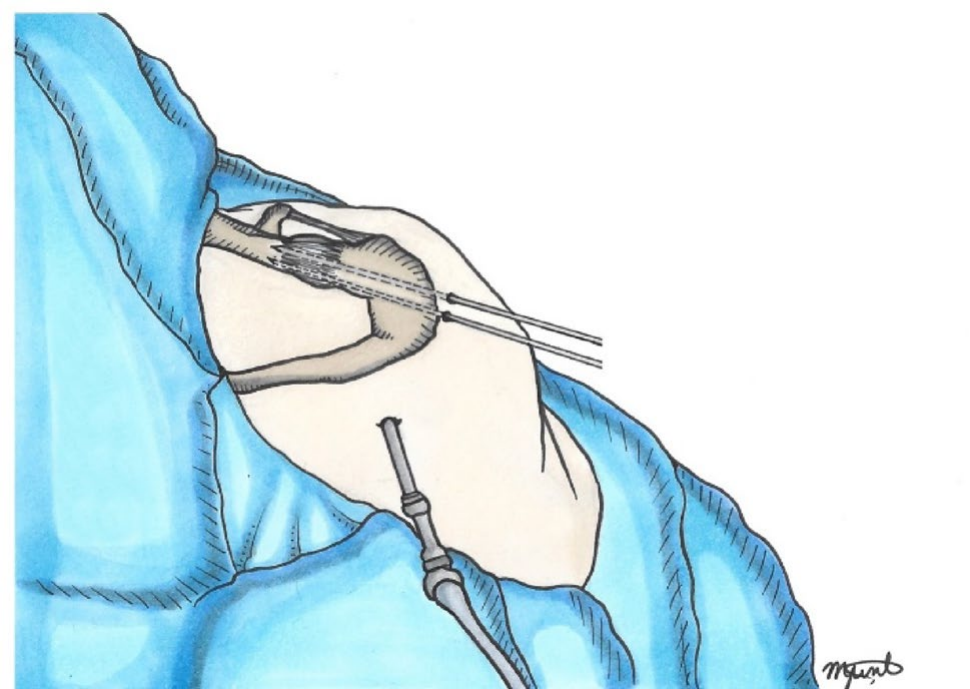
AC joint reduction. A 3-cm mini-open incision over the AC joint allow exposure and debridement. The joint is reduced anatomically and stabilized provisionally with Kirschner wires, confirmed under fluoroscopy

The ACJ is anatomically reduced, and it can be temporarily stabilized with two Kirschner wires after aligning their articular facets [4] (Fig. 2). Reduction is confirmed under fluoroscopic control.

### Harvest of the LHBT

The LHBT is released approximately 1 cm proximal to the musculotendinous junction. A subpectoral tenodesis is performed with a suture anchor, preserving elbow flexion and forearm supination strength, while maximizing graft length for reconstruction.

### Arthroscopic steps

Portals and Rotator Interval and Coracoid Exposure.

**Fig. 3:**
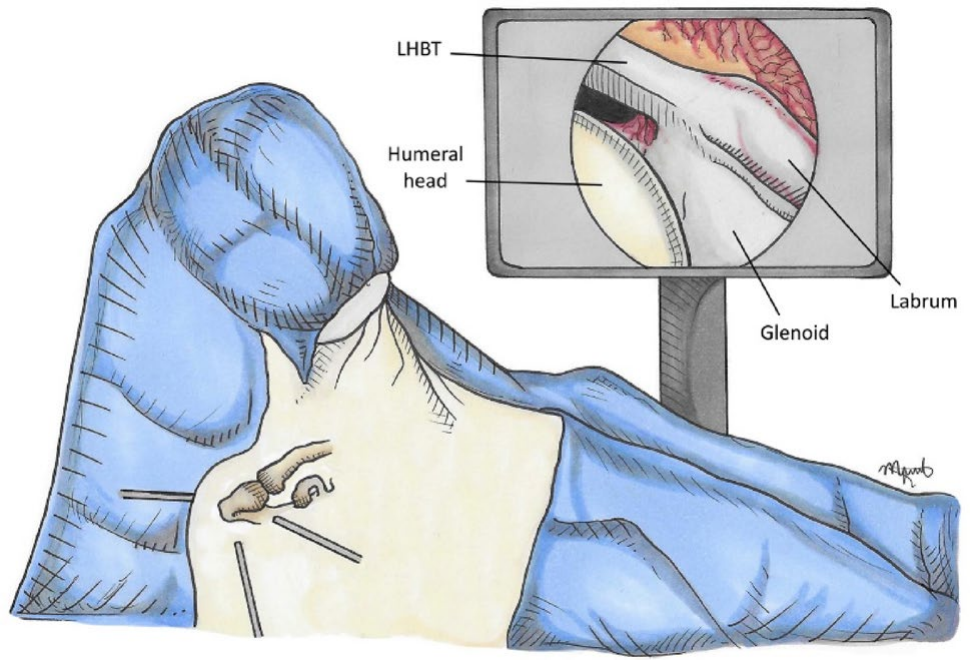
Surgical approach. Three arthroscopic portals allow the endoscopic-assisted procedure with inspection of the joint, secure the LHB tendon and opening of the rotator interval to visualize the coracoid base

Three standard arthroscopic portals are created: posterior, anterolateral, and anteromedial. The rotator interval is opened to visualize the coracoid base. The subcoracoid space is cleared, ensuring adequate visualization and working area (Fig. 3).

The proximal stump of the LHBT is redirected proximally, maintaining its native supraglenoid attachment. The tendon is delivered through the anteromedial portal, prepared extracorporeally with Krackow stitches on the free end using high-strength number two sutures, and the diameter is measured (typically 4–5 mm in diameter). The graft is trimmed and calibrated so that its diameter does not exceed 4.0 mm, ensuring compatibility with the clavicular tunnel.

### Coracoclavicular tunnel (elastic system passage)

A 2.4 mm guidewire is advanced from the superior clavicle, approximately 4.5 cm medial to the ACJ, toward the base of the coracoid under arthroscopic and fluoroscopic control. A drill guide system (Conmed Infinity ACL/PCL Femoral Footprint Guide Arm, Infinity Guide Body, Infinity Guide Sleeve Straight 2.4 mm – Conmed, Largo, FL, USA) is used at an angulation of 70–80°. A 2.0 mm hole is created after fluoroscopic confirmation, the suspensory fixation system (Infinity Button – Conmed, Largo, FL, USA) is passed and secured, using a suture passer (Super Shuttle – Conmed, Largo, FL, USA) to shuttle both suture strands, thereby restoring vertical stability.

### Clavicular Tunnel for the LHBT graft

A second tunnel is then prepared for the tendon itself, drilled in the clavicle more laterally than the suspensory system. This tunnel is placed around 3 cm medial to the ACJ and drilled to 4.0 mm diameter to allow passage of the LHBT graft. The prepared tendon is introduced with the aid of a suture shuttle, maintaining continuity with its native origin at the supraglenoid tubercle.

### Acromioclavicular cerclage and graft fixation

**Fig. 4:**
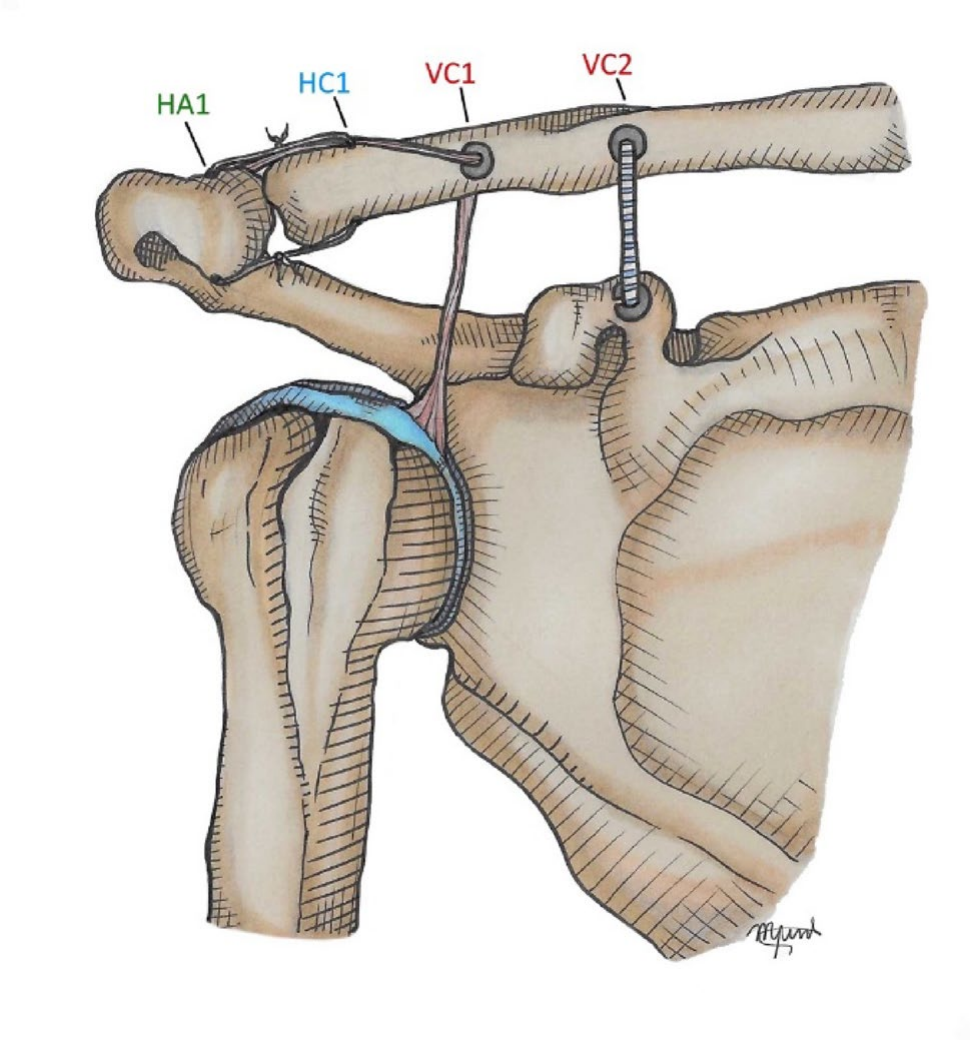
Coracoclavicular tunnel for suspensory fixation, AC cerclage and graft fixation. A 2.0 mm tunnel (VC2) is drilled from the clavicle to the coracoid base at 4.5 cm medial to the AC joint. The suspensory fixation system (Infinity Button) is passed **with a Super Shuttle device to shuttle both suture strands**, restoring vertical stability. A second tunnel (VC1) drilled 3–3.5 cm medial to the AC joint with a diameter of 4.0 mm, accommodates the calibrated LHBT graft. The graft is tensioned and cycled. Sutures from its free end are passed through a 2.0 mm clavicular tunnel (HC1) and a horizontal/oblique acromial tunnel (HA1), creating a cerclage that reinforces horizontal stability. The residual posterosuperior capsule and AC ligament are sutured and plicated to the graft to augment construct stability. HA1 – Horizontal Acromium 1; HC1 – Horizontal clavicle 1; VC1 – Vertical clavicle 1; VC2 - Vertical clavicle 2

Once the graft is retrieved above the clavicle, it is tensioned and cycled. An additional set of sutures placed 2–2.5 cm from the tendon extremity is passed through a horizontal 2.0 mm tunnel in the clavicle (2 cm medial to the ACJ) and then through a horizontal/oblique tunnel in the acromion (2 cm lateral to the ACJ). These sutures create a cerclage construct across the ACJ, reinforcing horizontal stability. The residual posterosuperior capsule and AC ligament are sutured and plicated to the graft to augment resistance and stability (Fig. 4).

### Verification and closure

Final joint stability is confirmed arthroscopically and fluoroscopically. Portals and the ACJ mini-open incision are closed in layers, and sterile dressings are applied.

### Anatomical evaluation

The primary outcome was the feasibility and reproducibility of the technique under endoscopic assistance. Secondary outcomes included anatomical accuracy of graft passage, correct positioning of fixation points, and absence of neurovascular conflict.

So, following completion of the procedure, specimen was carefully dissected to verify graft trajectory, tunnel positioning, and the relationship of the graft to adjacent neurovascular and capsuloligamentous structures to confirm reproducibility and anatomical safety. The key Pearls and Pitfalls of the technique are summarized in Table 1.

**Table 1:** Pearls and pitfalls of using the long head of the biceps (LHB) tendon as a pedicular autograft in AC joint reconstruction

Pearls	Pitfalls
Subpectoral LHBT tenodesis reduces the risk of common complications (loss of supination strength, Popeye deformity).	Oversized clavicular tunnels increase fracture risk; the tendon diameter should precisely match the tunnel diameter.
Dual vertical fixation points between clavicle and coracoid provide strong stability and enhances healing; if the elastic system fails, the LHBT still functions as a stabilizer.	The free LHBT end must be trimmed to ~ 4 mm diameter (over 4 cm length) to avoid enlarging the clavicular hole.
Secure fixation of the LHBT to the clavicle reinforces the ACJ.	Incorrect tunnel placement may compromise stability or injure neurovascular structures; fluoroscopic/endoscopic guidance is essential.
Cerclage of the ACJ with sutures attached to the LHB adds horizontal stability.	Excessive graft tensioning can over-constrain the joint, while insufficient tension may result in persistent instability.
The 4-mm clavicular tunnel is fully filled by the tendon, lowering the risk of fracture.	Inadequate tendon preparation or poor fixation may lead to graft loosening or failed reduction.
	Damage to the LHBT during mobilization compromises graft integrity and long-term stability.

## Discussion

The most important finding of this study was the demonstration—on cadaveric specimen—of the anatomical feasibility and technical reproducibility of using the LHBT tendon as a pedicular autograft for arthroscopic reconstruction of ACJ dislocation. This work builds directly upon our prior anatomical feasibility study performed through an open approach [21] and advances the concept by standardizing tunnel creation, graft passage, and fixation under arthroscopic visualization. The present study represents a critical step preceding clinical application, as it provides the opportunity to test and refine the procedure under controlled laboratory conditions using human anatomical specimens. Such preclinical validation is indispensable to anticipate technical challenges, minimizing the risk of complications, and optimizing reproducibility before implementation in patients.

The rationale for surgical treatment of AC joint dislocations is based on injury severity, chronicity, and patient-specific functional requirements. Surgical management of clinically significant acromioclavicular joint instability encompasses a wide range of open and arthroscopic techniques aimed primarily at restoring coracoclavicular stability using several strategies as Hook plate fixation [22], circumferential sutures cerclage techniques [15], synthetic suspensory devices [13], free tendon grafts [12], or combined constructs. Currently, beyond restoration of vertical stability, reinforcement of the acromioclavicular capsuloligamentous structures to restore horizontal stability is recommended [1–3, 15].

Biological augmentation of ACJ reconstructions with tendon grafts is recommended, particularly in chronic or high-grade injuries, to reduce the risk of recurrence and improve final outcomes [17]. Despite their widespread adoption, these approaches may alter native anatomy, rely on non-biological materials, or require graft harvesting with associated morbidity, and they do not consistently reproduce the complex anatomy and biomechanics of the coracoclavicular ligamentous complex. From an anatomical and biological perspective, there is increasing interest in reconstruction strategies that respect native tissue planes, preserve vascularity, and favor biological integration. In this context, the use of a pedicled LHBT autograft as described previously [21] represents an anatomically grounded alternative that leverages local tissue availability and orientation while minimizing additional surgical trauma. From a clinical perspective, minimally invasive arthroscopic techniques may provide natural advantages in terms of tissue preservation, surgical exposure, and the potential for a more anatomic and biologically favorable reconstruction.

Commonly used distant autografts, such as hamstrings, can add morbidity to an otherwise uninjured limb and prolong rehabilitation. By contrast, the LHBT autograft is adjacent to the injured structures, can be prepared and transferred through the same surgical field, and—because its origin at the supraglenoid tubercle is preserved—requires only one site of biological healing. The technique described not only addresses coracoclavicular stabilization but also permits reinforcement of the ACJ capsule and ligaments, thereby targeting both vertical and horizontal stability. These features, combined with the absence of lower-limb harvest, make the LHBT pedicled graft an appealing option for selected patients [21].

From a technical standpoint, several pragmatic advantages emerged during laboratory testing. Dual vertical fixation elements (elastic suspensory device plus the LHBT pedicled graft) create redundancy; should the elastic system fail, the LHBT continues to function as a stabilizer. Cerclage sutures linked to the LHBT contribute to horizontal control across the ACJ. Although a 4-mm clavicular tunnel is necessary, it is fully occupied by the graft when the free end is prepared to a 4-mm diameter over at least 4 cm, which may mitigate fracture risk.

## Limitations and disadvantages

Important limitations must be acknowledged. First, safe and effective execution of the procedure requires proficiency in both arthroscopic and open techniques, as tendon mobilization, tunnel creation, and fixation are technically demanding steps in which small inaccuracies can compromise the construct or increase the risk of complications. Second, LHBT tendon quality and diameter vary between individuals—particularly in older patients or those with degenerative changes—which may restrict applicability. Meticulous graft preparation is therefore essential: the tendon diameter should closely match the clavicular tunnel, and the free end should be tapered to approximately 4 mm over at least 4 cm to achieve a snug, load-sharing fit without enlarging the drill hole. A further limitation relates to the potential for tunnel widening. Even when the clavicular tunnel is initially drilled to a minimal diameter of 4 mm, progressive enlargement may occur postoperatively due to mechanical stress, micromotion of the graft, or the so-called “windshield-wiper effect.” Such tunnel widening has been associated with an increased risk of clavicular fracture in clinical series [11]. Therefore, although the present construct aims to minimize tunnel size, this complication must be acknowledged as it may compromise long-term stability and lead to structural failure.

Third, tunnel placement is critical. Misaligned or excessively large tunnels in the clavicle or coracoid may breach cortices or precipitate fracture. Techniques that involve transclavicular–transcoracoid drilling inherently carry these risks [14]. In our technique, we propose placement of the clavicular tunnel at 3 cm medial to the ACJ; however, no anatomical or biomechanical studies have yet determined the ideal position of such a tunnel relative to the ACJ. A practical advantage of the current construct is that, in the event of a coracoid fracture, the LHB tendon still provides continuity between the scapula (via its supraglenoid origin) and clavicle through the lateral tunnel, thereby preserving a stabilizing link and offering a built-in “rescue” configuration. Moreover, the technique can be executed without coracoid passage, if necessary, by routing the graft directly to the clavicle.

Another potential concern relates to the change in vector forces applied to the LHBT when it is redirected superiorly rather than along its native horizontal course. Such vertical loading could theoretically affect the stability of its anchorage and contribute to postoperative pain, given that the biceps tendon is richly innervated and a well-recognized source of tendinopathy and discomfort. Nevertheless, previous clinical studies evaluating procedures that incorporate the LHBT as reinforcement have not reported increased rates of biceps-related pain or complications [10, 23]. These findings suggest that, despite theoretical concerns, the use of the LHBT in reconstructive and augmentation procedures appears to be safe and well tolerated.

Potential complications also relate to the subpectoral LHB tenodesis [19] and to the ligamentoplasty across the clavicle, and acromion. While complications due to excessive proximal tension at the LHB origin have not been reported, they remain a theoretical concern that warrants careful intraoperative assessment of graft tension. The inclusion of limited open steps increases surgical exposure compared with fully arthroscopic methods and may modestly lengthen operative time.

Finally, this was a cadaveric pilot study. It does not provide biomechanical data under cyclic loading or clinical outcomes, and soft-tissue biology in cadaveric models cannot replicate healing in vivo. Rigorous biomechanical testing and prospective clinical studies are needed to confirm safety, reproducibility, durability of reduction, and patient-reported outcomes before widespread clinical adoption.

## Conclusion

This cadaveric investigation supports the concept that the LHBT can serve as a viable, anatomically favorable, and biologically advantageous pedicled autograft for ACJ reconstruction. The technique preserves the native supraglenoid origin, requires only one site of tendon healing, and eliminates at a distance donor-site morbidity associated with distant autograft harvest. Importantly, it provides both vertical and horizontal stabilization, thereby addressing the multidirectional nature of ACJ instability. Most significantly, this study describes an arthroscopic approach that enhances the accuracy of tunnel creation, refines graft passage, and reduces surgical morbidity compared with open procedures. This innovation not only consolidates the anatomical rationale for using the LHBT but also represents a meaningful step toward safer, more reproducible, and biologically sound reconstructions. Further biomechanical studies and prospective clinical trials are warranted to confirm its efficacy and long-term outcomes.

## Supplementary Information

Below is the link to the electronic supplementary material.

Supplementary Material 1

## Data Availability

No datasets were generated or analysed during the current study.
