# Supplementary material for: The long head of the biceps tendon as a pedicled autograft for acromioclavicular joint reconstruction: development of an arthroscopic technique
Source: Surg Radiol Anat. 2026 Mar 13;48(1):88. doi: 10.1007/s00276-026-03844-8 (PMC12987903; doi:10.1007/s00276-026-03844-8)
Supplement: Supplementary file 1 — Supplementary Material 1 [file 276_2026_3844_MOESM1_ESM.docx]

**The Long Head of the Biceps Tendon as a Pedicular Autograft for Acromioclavicular Joint Reconstruction: Development of an Arthroscopic Technique**

Nuno Sevivas^1,2,3,4^MD, PhD, Mariana Pinto^1,2^MS, Diogo Nunes Sousa^1,2^MS, Diogo Barreira^1,2^MS, Ana Catarina Ângelo^5,6^MD, Clara Azevedo^5,6^MD, PhD, Manuel Ribeiro da Silva^7^MD, PhD, Rui Claro^8^MD, PhD, João Espregueira-Mendes^1,2,9^ MD, PhD, Hélder Pereira^1,2,10,11^MD, PhD, Alexandre Lädermann^12,13,14,15^ MD, PhD

^1^ Life and Health Sciences Research Institute (ICVS), School of Medicine, University of Minho, Campus de Gualtar, 4710-057 Braga, Portugal.

^2^ ICVS/3B’s - PT Government Associate Laboratory, Braga/Guimarães, Portugal

^3^ S*houlder and Elbow Unit, Orthopaedics Department*, ULSAM Médio Ave, Famalicão, Portugal

^4^ S*houlder and Elbow Unit, Orthopaedics Department*, Trofa Saúde Hospital Braga Sul, Braga, Portugal

^5^ S*houlder and Elbow Unit, Department of Orthopaedic Surgery, Hospital dos SAMS de Lisboa, Lisbon, Portugal*

^6^ *Shoulder and Elbow Unit, Orthopaedics Department, Joaquim Chaves Saúde, Carcavelos, Portugal*

^7^ *Shoulder and Elbow Unit, Orthopaedic and Musculoskeletal Centre, Cuf Porto Hospital, Porto, Portugal*

^8^ S*houlder and Elbow Unit, Orthopaedics Department*, Centro Hospitalar Santo António, Porto, Portugal

^9^ *Clínica Espregueira Mendes*, Porto, Portugal

^10^ *Orthopaedics Department*, Centro Hospitalar Póvoa de Varzim / Vila do Conde, Póvoa de Varzim, Portugal

^11^*Ripoll y De Prado Sports Clinic:* Murcia-Madrid FIFA Medical Centre of Excellence, Madrid, Spain

^12^Division of Orthopedics and Trauma Surgery, La Tour Hospital, Meyrin, Geneva, Switzerland

^13^Faculty of Medicine, University of Geneva, Geneva, Switzerland

^14^Division of Orthopedics and Trauma Surgery, Department of Surgery, Geneva University Hospitals, Geneva, Switzerland

^15^Foundation for Research and Teaching in Orthopedics, Sports Medicine, Trauma, and Imaging in the Musculoskeletal System, Meyrin, Geneva, Switzerland

Corresponding author: **Nuno Sevivas**, MD, PhD

School of Medicine, ICVS/3B's - PT Government Associate Laboratory, University of Minho, Campus de Gualtar, 4710-057 Braga, Portugal.

Email: nunosevivas@med.uminho.pt; Phone: +351 253 604910

**Competing interests:**

- Alexandre Lädermann is a paid consultant for Arthrex, Stryker, and Medacta. He received royalties from Stryker and Medacta. He is the (co-)founder of FORE, Med4Cast, and BeeMed. He owns stock options in BeeMed and Follow Health. He is on the board of the French Arthroscopic Society.
- Other authors declare no conflicts of interest.

**Author contributions:**

**Nuno Sevivas (NS)** contributed to study conception and design. He also performed and interpreted the analyses and drafted the final manuscript. Moreover, he reviewed the work critically for important intellectual content.

**Mariana Pinto (MP)** contributed to the study conception and design. She was responsible for the images conception and execution. She also contributed for the interpretation of data work and revised the manuscript for important intellectual content.

**Diogo Nuno Sousa (DNS)** contributed to study conception and design. He also performed and interpreted the analyses and drafted the final manuscript. Moreover, he reviewed the work critically for important intellectual content.

**Diogo Barreira (DB)** contributed for the study design. He also contributed for the interpretation of data work and revised the manuscript for important intellectual content.

**Ana Catarina Ângelo (ACA)** contributed for the study design. She also contributed for the interpretation of data work and revised the manuscript for important intellectual content.

**Clara Azevedo (CA)** contributed for the study design. She also contributed for the interpretation of data work and revised the manuscript for important intellectual content.

**Manuel Ribeiro da Silva (MRS)** contributed for the study design. He also contributed for the interpretation of data work and revised the manuscript for important intellectual content.

**Rui Claro (RC)** contributed for the study design. He also contributed for the interpretation of data work and revised the manuscript for important intellectual content.

**João Espregueira-Mendes (JEM)** contributed for the study design. He also contributed for the interpretation of data work and revised the manuscript for important intellectual content.

**Hélder Pereira (HP)** contributed for the study design. He also contributed for the interpretation of data work and revised the manuscript for important intellectual content.

**Alexandre Lädermann** **(AL)** contributed to study conception and design. He also performed and interpreted the analyses and drafted the final manuscript. Moreover, he reviewed the work critically for important intellectual content.

**All authors agree to be accountable for all aspects of the work, approved the final manuscript for submission, and took the responsibility for the integrity of the work as a whole, from inception to finished article.**

Running Title: **LHBT Autograft for ACJ reconstruction**

**Funding**: None.
